# Aptamers: Functional-Structural Studies and Biomedical Applications 2.0

**DOI:** 10.3390/ijms25084279

**Published:** 2024-04-12

**Authors:** Romualdo Troisi, Filomena Sica

**Affiliations:** 1 Department of Chemical Sciences, University of Naples Federico II, 80126 Naples, Italy; romualdo.troisi@unina.it; 2 Institute of Biostructures and Bioimaging, CNR, 80131 Naples, Italy

As a follow-up to the previous Special Issue “Aptamers: Functional-Structural Studies and Biomedical Applications” [1], this second edition delivers numerous examples of the application of the oligonucleotide aptamers in different fields, from food chemistry to medicine, from drug delivery to infection monitoring. This broad versatility is the direct result of the numerous three-dimensional structures adopted by the aptamers, that enable them to recognize with high affinity and specificity various target molecules, from single ions to large molecular assemblies. A paradigmatic example is provided by the aptamers recognizing proteins, which display a variety of articulated architectures and, in some cases, experience intricate structural transitions from their protein-unbound to the protein-bound state. The complexity of these conformational changes requires both structural and computational approaches to be fully understood [2].

The properties and functions of the aptamers can be widely modulated and improved through chemical modifications and sequence variations. For example, the sequence mutation and extension of the yly12 aptamer lead to oligonucleotides showing improved binding affinities towards the sixth immunoglobulin-like domain of the L1CAM (L1 cell adhesion molecule), a protein highly relevant for human tumor formation, progression, and metastasis [3]. Similarly, the sequence of the most studied thrombin binding aptamer, named TBA, that adopts a two-layer G-quadruplex structure, was modified to produce three- and four-layer TBA analogues, which also contain non-natural alpha-deoxyguanosines at specific positions [4]. Despite the considerable increase in the thermodynamic stability of the resulting TBA analogues, they present a low anticoagulant activity against human thrombin. On the contrary, the conjugation of tripeptide sequences to these modified TBAs produce a recover and improvement of their anticoagulant activity [4].

Lately, TBA is receiving increasing attention in the development of electrochemical aptasensor for target analytes detection. In particular, the application of TBA for the monitoring of Pb^2+^ and, mostly, Sr^2+^, that can contaminate food and drinks, has been studied by theoretical methods [5]. Indeed, these bivalent cations induce in the G-quadruplex structure of the aptamer small but significant changes, which modify the electrical responses of the sensor [5]. In a separate investigation, the determination of trace Pb^2+^ in food and water samples was successfully achieved by a three-mode biosensoring assay platform, in which a highly sensitive catalytic amplification indicator, CB@AgNPs (silver nanoparticles loaded in cholesteryl benzoate liquid crystals), has been combined with a highly selective aptamer [6].

Aptamers are also successfully exploited in the development of biosensors for the rapid, on-site, real-time, and cost-effective monitoring of toxins, bacteria, viruses, etc. For example, for the detection in food of fumonisin B_1_ (FB_1_), a highly toxic and abundant carcinogenic mycotoxin, an aptasensor was developed using a nuclease-triggered signal enhancement and graphene oxide as a fluorescence quenching agent towards a fluorophore-modified FB_1_ aptamer [7]. Furthermore, an aptamer library specific for *Roseburia intestinalis* bacterium, whose changes in its intestinal abundance can cause obesity, enteritis, and atherosclerosis, has been selected, containing potential candidates for the construction of electronic biosensors to be used in the monitoring of *R. intestinalis* in gut microbiomes [8]. Promising aptasensors able to identify Dengue, Zika, and SARS-CoV-2 viruses in complex clinical samples have been inspired by the need for rapid monitoring tools to prevent the transmission of the viral infections. In particular, the detection of the flaviviruses Dengue and Zika has been achieved by identifying a bifunctional aptamer, named APTAZC10-MB, capable of capturing, directly in the serum sample, the viral RNA, which is subsequently amplified by reverse transcription followed by polymerase chain reaction (RT-PCR) [9]. To detect the spike glycoprotein and, consequently, the viral particles of three variants of the coronavirus SARS-CoV-2, an electrochemical impedance spectroscopy-based aptasensor has been developed functionalizing an interdigitated gold electrode surface with the CoV2-RBD-1C aptamer supplemented with a hexaethylene glycol spacer [10]. Another electrochemical aptasensor, based on square wave voltammetry, has been developed for the detection of the humanized therapeutic antibody bevacizumab using an anti-idiotype bivalent aptamer modified with a redox probe [11].

In the effort to create advanced biosensors, particularly interesting is the case of the light-up aptamers, RNA oligonucleotides that bind with their cognate fluorogen ligands and activate their fluorescence. For instance, by splitting the light-up Broccoli aptamer has been generated a system able to activate the emission signal of the fluorophore DFHBI-1T exclusively in the presence of a complementary sequence [12]. Notably, the potential application of this approach is not only the in vitro/in vivo detection of different DNA/RNA targets, but also the real-time monitoring of the self-assembly of nucleic-acid-based devices in vivo and of the intracellular delivery of therapeutic nanostructures [12].

Overall, the papers included in this second Special Issue confirm the high impact of the aptamer application in diverse areas within analytical chemistry, biotechnology, biomedicine, and molecular biology. The outlook for aptamer tools right now looks really good and they are expected to succeed in addressing several basic and applied scientific challenges.
